# Can Ultrasound Therapy Be an Environmental-Friendly Alternative to Non-Steroidal Anti-Inflammatory Drugs in Knee Osteoarthritis Treatment?

**DOI:** 10.3390/ma14112715

**Published:** 2021-05-21

**Authors:** Rodica Ana Ungur, Viorela Mihaela Ciortea, Laszlo Irsay, Alina Deniza Ciubean, Bogdana Adriana Năsui, Răzvan Andrei Codea, Victoria Emilia Singurean, Oana Bianca Groza, Simona Căinap, Georgiana Smaranda Martiș (Petruț), Cristin Borda, Ileana Monica Borda

**Affiliations:** 1 Department of Medical Specialties, Faculty of Medicine, “Iuliu Hatieganu” University of Medicine and Pharmacy, 400012 Cluj-Napoca, Romania; ungurmed@yahoo.com (R.A.U.); laszlo.irsay@umfcluj.ro (L.I.); alina.deniza.ciubean@gmail.com (A.D.C.); tuns.victoria@umfcluj.ro (V.E.S.); grozaoanabianca@yahoo.com (O.B.G.); monica.borda@umfcluj.ro (I.M.B.); 2 Department of Community Health, Faculty of Medicine, “Iuliu Hatieganu” University of Medicine and Pharmacy, 400012 Cluj-Napoca, Romania; adriana.nasui@umfcluj.ro; 3 Faculty of Veterinary Medicine, University of Agricultural Science and Veterinary Medicine, 400372 Cluj-Napoca, Romania; razvan.codea@usamvcluj.ro (R.A.C.); cris_mv@yahoo.com (C.B.); 4 Department of Mother and Child, Faculty of Medicine, “Iuliu Hatieganu” University of Medicine and Pharmacy, 400012 Cluj-Napoca, Romania; sorana.cainap@umfcluj.ro; 5 Food Engineering Department, University of Agricultural Sciences and Veterinary Medicine Cluj-Napoca, 400509 Cluj-Napoca, Romania

**Keywords:** non-steroidal anti-inflammatory drugs, ultrasound therapy, health benefits, environment pollutants

## Abstract

The non-steroidal anti-inflammatory drugs (NSAIDs) are the most used drugs in knee osteoarthritis (OA) treatment. Despite their efficiency in pain and inflammation alleviation, NSAIDs accumulate in the environment as chemical pollutants and have numerous genetic, morphologic, and functional negative effects on plants and animals. Ultrasound (US) therapy can improve pain, inflammation, and function in knee OA, without impact on environment, and with supplementary metabolic beneficial effects on cartilage compared to NSAIDs. These features recommend US therapy as alternative for NSAIDs use in knee OA treatment.

## 1. Introduction

In the absence of disease-modifying treatment, the burden of osteoarthritis (OA) is increasing globally. The ageing and the increasing of obese population make this syndrome more prevalent than in previous decades [1]. OA showed a significant increase of 31.4% from 2007 to 2020, after another significant increase of 63.1% between 1990 and 2007 [2]. Approximately 85% of the burden of OA worldwide is connected with knee OA [3]. The prevalence of knee OA increased significantly over the last decades and continues to rise [4]. For knee OA, in 2020, the global prevalence was 16.0% (95% confidence interval (CI), 14.3–17.8%) and global incidence was 203 per 10,000 person-years (95% CI, 106–331) [5]. However, an ideal treatment for knee OA does not exist at this time [6].

## 2. NSAIDs–Most Used Medication in Knee OA

Main knee OA symptoms include pain, reduced function, stiffness, and joint instability. Patients may also experience movement decreasing, deformity, swelling, and crepitus. When pain persists, patients develop pain-related psychological distress [7] and are exposed to addictive behaviors and poor evolution of their comorbidities [8,9,10,11]. The OA symptoms are caused by a complex pathology of whole joint, generated by inflammation; oxidative stress; and metabolic, endocrine, and genetic factors. All that induces chondrocytes death, imbalance between repair and destruction process of cartilage, hypertrophy, and increased vascularization in synovial membrane, bone turnover increase, and osteophytes occurrence.

The non-steroidal anti-inflammatory drugs (NSAIDs) are amongst the most popularly used drugs in OA, due to the efficacy in reducing inflammation and pain [12]. Until recently, OA guidelines included paracetamol and NSAIDs for pain relief, but in 2017, a meta-analysis concluded that the very small effect size of paracetamol compared to placebo does not recommend it as single agent in the treatment of knee and hip OA [13]. One year later, topical NSAIDs were shown to be effective for pain and function relief in OA compared to placebo [14], and in 2019, Osteoarthritis Research Society International guidelines for the non-surgical management of knee, hip, and polyarticular osteoarthritis (OARSI) recommended topical NSAIDs as first line in knee OA, and cyclooxygenase (COX)-2 inhibitors or NSAIDs associated with proton pump inhibitors for individuals with gastrointestinal comorbidities [15]. In the guidelines, there is no other drug recommended as alternative for NSAIDs. Acetaminophen/paracetamol was conditionally not recommended, and oral and transdermal opioids were strongly not recommended [15].

The numerous prescriptions for patients with rheumatoid arthritis [16], ankylosing spondylitis [17], cancer pain [18], or infectious febrile syndromes [19], as well as those for veterinary pathology, where joint pain and functional limitations benefit also from NSAIDs treatment [20,21,22], are added to the prescriptions of NSAIDs for OA patients.

## 3. NSAIDs as Contaminants of the Environment

Unfortunately, in spite of their beneficial effect, NSAIDs are now considered as emerging contaminants of the environment [23]. In an excellent article, Tyumina presented the detrimental effects of NSAIDs for the environment, starting from their partial resistance to metabolization and inactivation, and ending with their entry into the food chain of living organisms. Among the NSAIDs, diclofenac, ibuprofen and naproxen are the most recurrently detected in the environment and recognized as potential emerging contaminants with high octanol–water partition coefficient and low acid dissociation constant values [24].

NSAIDs arrive in the ambient environment with industrial and municipal wastewater because they are not completely metabolized in the human body [25], and pre-treatment for inactivation of expired and defective NSAIDs is not strictly controlled and completely efficient [26].

A significant percentage of NSAIDs are excreted through urine and feces and enter in the municipal wastewater. Naproxen is eliminated in its unchanged form (10%) or as metabolites (60%) through the urine and feces [27]. The average percentage of excretion for diclofenac is 6–39%, and for ibuprofen is 5–15%, as an unchanged form or as metabolites [28,29].

In the United States of America (USA), ibuprofen and naproxen were the most frequently NSAIDs detected in surface water, with average detection frequency ranging from 18% to 33%.

In sampled water source, ibuprofen was found in concentrations greater than 1000 ng/L, with the maximum concentration observed greater than 1500 ng/L, and ibuprofen exceeded 100 ng/L in drinking water [30]. Recently Ibuprofen was included in contaminants of emerging concern (CECs), found in the water cycle in the USA, selected by criteria that included usage, occurrence, resistance to treatment, persistence, and physicochemical properties relevant for the potential degradability of a class of compounds. CECs represent the most persistent compounds in surface waters and detected with significant frequency throughout the water cycle [30].

Naproxen, the most utilized NSAID for OA patients in USA, was detected in all types of water, including drinking water and groundwater. Although the concentrations that have been observed in USA were low, from ng/L to μg/L, the long-term exposure to these concentrations may have a negative effect on living organisms, especially when naproxen is mixed with other drugs [31]. Unfortunately, in Europe, naproxen concentrations exceeded 2.027 μg/L in 69% of water samples harvested from more than 100 European rivers [32].

Diclofenac was detected in surface water, groundwater, and/or tap/drinking water in 50 countries and was the most frequently detected pharmaceutical in environmental samples globally. In surface waters from 12 countries worldwide, the weighted average concentrations exceeded the predicted no-effect concentrations of 0.1 μg/L, indicating an unacceptable risk for the environment [33].

Naproxen, diclofenac, and ibuprofen, next to paracetamol and acetylsalicylic acid, are included now in the list of substances that were detected in surface, drinking, and groundwater in all United Nations regional groups (Africa Group, Asia-Pacific Group, Eastern Europe Group, Latin American and Caribbean States Group, and Western Europe and Others Group, which includes North America, Australia, and New Zealand), with global average concentrations ranging between 0.032 and 0.922 μg/L [27,33].

Among the frequently studied NSAIDs, ibuprofen, naproxen, diclofenac, and ketoprofen have similar structure (aromatic rings and carboxylic acid) and limited solubility that confer their resistance to biological treatments such as conventional activated sludge, trickling filters, and moving bed biofilm reactors [30,34,35].

These features allow the bioaccumulation of NSAIDs in freshwater and marine water bodies, surface waters, wastewater, groundwater, and even in drinking water, at concentrations ranging from a few ng/L up to several μg/L [36].

NSAIDs were also present in mollusks, fish, and crustaceans. NSAIDs present in the aquatic environment pass in the effluents from the wastewater treatment plants and into source water for animals and drinking water. The accumulation of NSAIDs in soil systems is facilitated by application of sewage sludge (biosolids) and increasing reclaimed wastewater use for irrigation in plants crops [37]. NSAIDs from water may be absorbed into aquatic organisms. In a lake in which diclofenac and ibuprofen were present as water pollutants, it could be found also in the bile of roach and wild bream [38].

Due to their resistance to biodegradation, NSAIDs are able to enter into food chains and pass from aquatic to soil bacteria [39], terrestrial invertebrates and vertebrates.

## 4. The NSAIDs Ecotoxicity

The NSAIDs ecotoxicity derived from their high reactivity and stability and their resistance to biodegradation. Moreover, not only NSAIDs but also their metabolites are globally entering the environment. For diclofenac, photo-transformation metabolites are even more toxic than diclofenac, and in algal reproduction tests three of diclofenac metabolites exhibited a six-fold increase in toxicity [40]. Similar results were reported for naproxen, its photo-derivatives being reported as more toxic for numerous species of bacteria (*Vibrio fischeri*), rotifer (*Brachionus calyciflorus*), and crustaceans (*Thamnocephalus platyurus*, *Daphnia magna*, and *Ceriodaphnia dubia*) [31,41,42].

In the ranking for pharmaceutical compounds conceived by Besse and Garric in 2008, according to the risk for the environment, ibuprofen and diclofenac are classified as Class IA and IIB respectively. In this ranking, Class IA included the compounds with the highest risk, and Class IV included those with only a very low risk [43]. Similarly, environmental risk assessment for active pharmaceutical compounds in surface waters, based on ‘measured environmental concentration’ and ‘predicted no effect concentration’, indicated that ibuprofen and diclofenac pose a high risk to aquatic ecosystems [44]. On the other hand, the investigations of European Union waters have found that concentration of naproxen in surface waters and in wastewater treatment plants exceeded by 10- to 500-fold the concentration recommended by the European Medicines Agency [45].

The plants, algae, invertebrates, and vertebrates exposed to NSAIDs develop oxidative stress (OS), metabolic perturbations, and changes in gene expression.

### 4.1. NSAIDs Toxicity for Plants

Metabolites resulting from NSAIDs in humans are excreted and can reach the wastewater treatment plants. On the other hand, at the end of the wastewater treatments, a fraction of NSAIDs or NSAIDs metabolites may be found in the biosolids, solid, or semisolid residues. Due to their high organic matter content, these subproducts may be used to improve the physical conditions of soils [46]. The NSAIDs present in contaminated biosolids can be degraded or absorbed in the surface soil layers, leach into ground water, or runoff into surface water [39]. In Algerian wastewater influent/effluent, NSAIDs were detected, with concentrations ranging from 155.5 to 6554 ng/L, demonstrating an incomplete removal efficiency of wastewater treatment plants, between 30.3 and 95% [47]. Ibuprofen can be removed by wastewater treatment plants up to 80%, but a significant amount remains in the effluents. Thus, the use of these reclaimed waters for irrigation, together with repeated application of sewage sludge, can lead to accumulation of ibuprofen in agricultural areas [48,49].

Irrigation with reclaimed wastewater and land application of biosolids, as well as ground water or surface contaminated water, introduce NSAIDs and their metabolites into agricultural environments, including plants cultures [50].

In vegetables experimental model, the exposure of orache (*Atriplex patula* L.), spinach (*Spinacia oleracea* L.), and lettuce (*Lactuca sativa* L.) seeds to diclofenac, ibuprofen, and naproxen, at concentrations of 0.1 mg/L, 0.5 mg/L and 1 mg/L, respectively, induced a moderate reduction of foliage physiological activity and the increase of emission rates for the 3-hexenal and monoterpene. After NSAIDs exposure, a moderate reduction in the assimilating pigments, total polyphenol, and flavonoid contents in these vegetables was noticed. Microscopic analysis showed that NSAIDs induced irregular growth of glandular trichomes on the surface of the adaxial side of the leaves, less stomata, and cells with less cytoplasm [51,52].

Cowpea plants (*Vigna unguiculata* L.) experimentally exposed to ibuprofen developed morphological changes including reduced shoot and root lengths, and leaf area and also OS and composition changes: decreased content for carotenoid, total chlorophyll and soluble protein, and impaired mineral balance [49].

The bean (*Phaseolus vulgaris* L.) treatment with aqueous diclofenac solutions (0.1, 0.2, 0.3, and 0.4 g/L) exhibited decreased values of net assimilation rates and stomata conductance to water and increasing of stress markers (Z)-3-hexenol and monoterpenes [53].

The meclofenamic acid and flufenamic acid, belonging to the fenamic acid class of NSAIDs, had also detrimental effects on plants development.

In the Arabidopsis model plant, 10 μM meclofenamic acid and 40 μM flufenamic acid treatments led to shorter and agravitropic primary roots and inhibited lateral root organogenesis through inhibitory effect on the chaperone activity, actin cytoskeleton dynamics, and endosomal trafficking [54]. Impacts upon higher plants are compound specific and differ between plant species. In experimental models, 10 μg/L compounds from the fenamic acid class affected *R. sativus* root endpoints (root length and water content), while ibuprofen affected early root development of *L. sativa* [55].

### 4.2. NSAIDs Toxicity for Invertebrates and Vertebrates

At low, environmentally relevant concentrations, NSAIDs have sub-lethal effects and hazardous consequences to the whole freshwater trophic chain [27].

Although in fresh waters the concentration of NSAIDs can be considered as relatively low, their high biological activity may induce a serious risk for non-target organisms, including freshwater invertebrates. Chronic toxicity of diclofenac in freshwater invertebrate species includes proteotoxicity [56], cyto-genotoxicity, destabilization of the lysosomal membrane [57], and immunotoxicity [58]. At low, environmental concentrations, ibuprofen can imbalance the oxidative status of mussels and provoke the onset of OS in freshwater invertebrates [59]. Chronic exposure to naproxen can inhibit the reproduction of crustaceans *D. magna* and *M. macrocopa* [60] and increase lipid peroxidation in freshwater mussels *Elliptio complanata* [61].

Among invertebrate marine organisms, *Mytilus galloprovincialis* exposed to diclofenac, ibuprofen, and ketoprofen at a realistic environmental concentration of 2.5 μg/L for up to 60 days developed genotoxic effects, alterations of immunological parameters, lipid metabolism, and changes in cellular turnover [62].

In non-target vertebrates NSAIDs cause genetic, enzymatic, and organ damages. Inhibition of the function of antioxidant enzymes catalase (CAT) and glutathione peroxidase after the fish exposure to ibuprofen or diclofenac at low concentration (0.2–60 μg/L) was observed in a study published by Stancova [63]. Rainbow trout (*Oncorhynchus mykiss*) exposed to environmentally relevant concentrations of at least 5 μg/L diclofenac revealed kidney damages such as a hyaline droplet degeneration in the tubular epithelial cells and the occurrence of interstitial nephritis. At the same concentration, necrosis of pillar cells of the fish occurred, leading to damage of the capillary wall within the secondary lamellae in the gills [64]. Even at 1 μg/L, diclofenac accumulated in kidney, liver, muscle, and gills tissues of the rainbow trout and caused cytological alterations [65]. Diclofenac accumulation in the environment caused renal impairment and a tragic decline (>95%) in the vulture population of Pakistan [66].

Chronic toxicity of naproxen evaluated on Japanese rice fish (*Oryzias latipes*) revealed that naproxen in concentrations of 0.5 mg/L significantly decreased the survival of juvenile fish and significantly increased transcription of ERβ2 gene [60]. The subchronic exposure to naproxen sodium and naproxen sodium mixture with tramadol hydrochloride had also a strong effect on OS, hatching, developmental rate, morphology, histopathology, and, in the case of mixture, mortality of common carp (*C. carpio*) in the early life stages [67]. After adult zebrafish (*Danio rerio*) exposure to naproxen at the environmental concentration of 1 μg/L, for 2 weeks, the messenger ribonucleic acid (mRNA) level of Ucp-2, involved in the control of mitochondria-derived reactive oxygen species, decreased, and the mRNA level for the antioxidant enzyme CAT was upregulated significantly, without significant effect on the same genes in the liver [68]. On the other hand, Zebrafish (*Danio rerio*) embryos and larvae were more sensitive to acute (8-day) toxicity, developing hatching inhibition, lower heart rate, pericardial edema, and histopathological liver damage, including hepatocytes vacuolarization and nuclei pycnosis [69].

Despite the NSAIDs concentrations detected in groundwater in most countries being under the potential environmental significant level, this should not eliminate the concern and the attempt to reduce the use of NSAIDs. Now, it is demonstrated that in surface water the COX inhibitors, a subclass of NSAIDs, may influence aquatic organisms more than was expected based on NSAIDs concentration [70]. Moreover, recent studies have proposed to decrease the upper limits for this group of drugs in drinking water [24].

## 5. US as Alternatives for NSAIDs in OA Treatment

Because the voluntary reduction of NSAIDs intake or their effective neutralization are problems far from being solved, it is necessary to find urgently alternative means of treatment, environment friendly, able to stop inflammation, pain, and degeneration of joint cartilage in OA patients.

In the last years, many non-drug therapeutical approaches for knee OA have been studied. Numerous trials proved the beneficial effects of exercise on pain and joint function in knee OA patients [71,72], with minimal risk of negative consequences and additional benefits on muscle structure and function, body composition, psychological health, sleep, fatigue, quality of life [73,74], and without environmental pollution.

Other non-drug therapeutic methods found to efficiently reduce pain and improve function in knee OA were intermittent pneumatic compression [75], manual therapy, use of braces, orthosis or canes, psychosocial interventions, education, weigh loss [76,77], yoga, acupuncture, radiofrequency ablation, thermal modalities [78], mud-pack therapy, mud-bath therapy, balneotherapy, and spa therapy [79].

In recent years, a large number of studies assessed the clinical and structural effects of physiotherapy-transcutaneous electrical nerve stimulation (TENS), laser therapy, ultrasound therapy, pulsed electromagnetic fields, and pulsed shortwave therapy and demonstrated analgesic, anti-inflammatory, anti-apoptotic, and matrix protective effects on joints [80,81,82,83]. These effects let us hope that physiotherapy agents can become a viable alternative to the use of NSAIDs, at least for certain periods of time.

Previous studies demonstrated the efficacy of pulsed electromagnetic fields on improving physical function in knee OA, but results on pain alleviation were contradictory [84,85,86].

Among the physiotherapy procedures, US therapy meets the efficacy and safety criteria that recommend it as a viable alternative to the use of NSAIDs in OA.

Along with the clinical effects of relieving pain and improving joint function, US therapy is able to improve all the three major events responsible for the OA progression: joint inflammation, chondrocyte death, and degradation of extracellular matrix (ECM).

An additional advantage is that US may be indicated in a very large number of patients, including elderly patients and patients with cardiovascular, renal, hepatic, or gastric diseases, in which NSAIDs may be limited or contraindicated. The number of contraindications for US therapy is extremely low, and systemic effects are absent at the frequencies and intensities used in OA treatment.

In clinical trials, US therapy for OA has been shown to be effective, as single treatment or combined with exercise or another physical agent that is also free of environment pollution effects.

### 5.1. US Therapy Improves Joint Function and Alleviates Joint Pain in OA Patients

Previous studies have shown that US, as isolated therapy or associated with physical activity or education, might be beneficial for pain relief and function improvement in patients with knee OA.

Several systematic reviews and meta-analysis have been published to date and have analyzed the effectiveness of US therapy in various administration variants in patients with knee OA: continuous US, low-intensity ultrasound (LIUS), and low-intensity pulsed ultrasound (LIPUS), all in nonfocused application and focused low-intensity pulsed ultrasound (FLIPUS).

Studies published to date have shown a reduction in joint pain in patients with knee OA after nonfocused US therapy in continuous mode, at a frequency of 1 MHz and intensities between 0.1 and 2.5 W/cm^2^ [87,88]. Significant reduction in pain and improvement in joint function have also been demonstrated in patients with knee OA after nonfocused US therapy in pulsed application at frequencies of 1, 1.5 and 3 MHz and intensities between 0.1 and 2.5 W/cm^2^ [87,88,89].

In knee OA patients, FLIPUS, applied at low frequency of 0.6 MHz, at low intensity of 0.12 W/cm^2^, in a focused mode, with pulse repetition frequency of 300 Hz and a duty cycle of 20%, showed additional benefits, improving not only pain and functional scores but also gait speed and most items of health-related quality of life assessed by the Medical Outcomes Study 36-item short-form health survey (SF-36). FLIPUS therapeutic effects on pain, recorded on the visual analogue scale and knee function, assessed by Lequesne index, were also evident at both 4- and 12-weeks follow-up [90]. In the meta-analysis published by Wu in 2019 phonophoresis US (US + NSAID) was not found superior to conventional non-drug US in effectively improving pain relief [91].

In terms of safety, no occurrence of adverse events caused by US therapy was reported in any trial that analyzed US therapy in short-duration of application (5–20 min daily), at low frequency of 0.6–1.5 MHz and intensity between 0.1 and 2.5 W/cm^2^ [88,89,90], but also for long-duration of application (4 h daily, for 6 weeks) of US therapy, at high frequency of 3 MHz and low-intensity of 0.132 W/cm^2^ [92].

### 5.2. US Beneficial Effects on OA Pathogenic Mechanism

The clinical human studies on US beneficial effects on OA pathogenic mechanism are few but concordant with studies on animal experimental OA, cartilage explants, or chondrocyte cultures. LIPUS applied at low intensity (<1 W/cm^2^) and low frequency (<1 MHz) was found efficient in alleviating joint swelling and inflammation in OA patients [93]. In continuous mode, US had antioxidant effects in OA patients [94]. For OA patients, US therapy administered at 1 MHz frequency with a peak intensity of 1 W/cm^2^, at a 20% duty cycle, led to increase in medial tibia cartilage thickness [95].

### 5.3. Anti-Inflammatory and Antioxidant Effects of US in Experimental Models

#### 5.3.1. US Counteracted Effects of Interleukin (IL)-1β

Wang demonstrated very recently that LIPUS at 100 mW/cm^2^ intensity, 1 MHz frequency, pulsed repetition frequency 100 Hz, and duty cycle 20% counteracted effects of IL-1β on chondrocytes by activation of zinc exporter-9 that downregulated ECM-degrading enzymes (Matrix metalloproteinase (MMP)-3, ADAMTS-5, and ADAMTS-8) and metal regulatory transcription factor-1 and upregulated aggrecans [96]. In porcine OA temporomandibular chondrocytes, LIPUS at frequency of 3 MHz, intensity of 30 mW/cm^2^, and with pulsing at 20% (2 ms on and 8 ms off) suppressed COX-2 mRNA level that was up-regulated by the treatment with IL-1β. For this effect, LIPUS activated integrin β1 receptor, followed by phosphorylation of ERK 1/2 [97]. US (1.5 MHz frequency and 30 mW/cm^2^ intensity) treatment of engineered 3D neocartilage construct exposed to IL-1β increased the glycosaminoglycans content and downregulated the mRNA level of MMP-1 [98].

#### 5.3.2. US Suppressed Tumor Necrosis Factor (TNF)-α Synthesis and Its Effects

Continuous LIUS, at 300 mW/cm^2^ intensity, 1 MHz frequency, suppressed TNF-α synthesis in OA cartilage in rabbit [99]. In bovine osteochondral explants with chondral fissures, continuous LIUS at frequency of 5 MHz, intensity <20 mW/cm^2^, at a constant pressure amplitude of 14 kPa, at input voltage of 2.5 Vpp (peak-to-peak voltage), suppressed TNF-α-induced increase in nuclear factor kappa-light-chain-enhancer of activated B cells (NF-κB) expression and upregulated the expression of collagen II and tissue inhibitor of metalloproteinase-1 genes [100].

#### 5.3.3. US Decreased NO and COX-2 Expression and Improved ECM Structure

LIPUS at 1.5 MHz frequency, 30 mW/cm^2^ intensity, increased COX-2 expression via integrin/integrin-linked kinase/protein kinase B (Akt)/NF-κB and p300 signaling pathway in human cultured chondrocytes [101]. In human OA explants, COX-2-dependent PGE_2_ modulates cartilage proteoglycan degradation [102]. In rabbit experimental OA, LIPUS (frequency 800 ± 5% KHz, intensity 50 ± 10% mW/cm^2^) inhibited secretion of NO, reduced expression of MMP-3, 7, 13, and promoted synthesis of collagen type II and proteoglycan in OA cartilage [103]. In the same experimental condition, FLIPUS, at frequency of 0.6 MHz, intensity of 120 mW/cm^2^, and a duty cycle of 20%, reduced PGE_2_ and NO synthesis in synovial fluid and attenuated release of type II collagen and proteoglycans, concomitantly with downregulation of chondrocyte apoptosis [104].

#### 5.3.4. US Effects on Viability and Metabolism of Chondrocytes

Studies on human chondrocytes have shown the ability of LIPUS to stimulate chondrocyte proliferation at a frequency of 1.5 MHz and intensity of 30 mW/cm^2^ [105], and at a frequency of 1 MHz and intensity of 67 mW/cm^2^ [106].

US in continuous mode increases cell viability at frequencies of 1.5 MHz and intensities below 30 mW/cm^2^ [107] causes mitochondrial and reticular proliferation and increases cellular metabolism at frequencies of 1 MHz and intensity of 200 mW/cm^2^ [108].

The effects are more obvious in OA cartilage compared to normal cartilage, in the deep layers compared to the superficial ones [105] and at younger ages [109].

#### 5.3.5. Effects of US on the ECM

LIUS acts directly on chondrocytes via stress-activated ion channels and integrins, increasing intracellular Ca^2+^ and modulating the phosphatidylinositol 3 kinase (PI3K)/Akt pathway and the mitogen-activated protein kinase (MAPK) signaling pathway, last consisting of ERK, p38 MAPK (p38), and c-Jun N-terminal kinase (JNK) [110]. LIPUS at frequency of 3 MHz and intensity of 40 mW/cm^2^, 20% on–off ratio, promoted cartilage repair in rabbit OA cartilage by downregulation of MMP-13, ERK1/2, and p38 [111]. It has been shown that LIPUS at frequency of 1 MHz and intensity of 48 mW/cm^2^ stimulated the synthesis of type II collagen and aggrecans in human child chondrocytes [109]. In continuous mode US, at 1 MHz frequency and 200–300 mW/cm^2^ intensity, it stimulated also synthesis of type II collagen and proteoglycans in human articular chondrocytes, in parallel with inhibition of MMP-1 synthesis [108]. mRNA expression of type II collagen was enhanced by LIPUS (intensity of 30 mW/cm^2^) in rat chondrocytes [112]. LIPUS (intensity of 30 mW/cm^2^, frequency of 1.5 MHz with a 200-μs tone burst repeated at 1.0 KHz) promoted proliferation of pig cultured chondrocytes and synthesis of type-IX collagen [113].

## 6. US Therapy Versus NSAIDs Treatment

To date, there are a small number of studies that compare US therapy with NSAIDs, and they are focused only on the topical administration of NSAIDs.

Ibuprofen phonophoresis with US at 1 MHz frequency, 1 W/cm^2^ intensity, and ibuprofen cream containing 5% ibuprofen were found not significantly different in pain and function improvement rates compared to US therapy at the same parameters, administrated alone, in knee OA [114], but one study communicated that the ibuprofen gel phonophoresis improved pain and Western Ontario and McMaster Universities (WOMAC) physical function score better than the ibuprofen cream phonophoresis [115].

US therapy, at 1 MHz frequency and 1.5 W/cm^2^ intensity, had similar effectiveness as phonophoresis with topical gel containing 1.16% diclofenac diethylamonium on pain and physical activities improvement in knee OA patients, immediately after treatment and at 3-month follow-up period, except walking duration, when phonophoresis was more successful [116].

In a previous study, diclofenac gel phonophoresis had similar efficacy on pain and functional status improvement in patients with knee OA for both continuous and pulsed mode (20% duty cycle) of US therapy (at 1 MHz frequency and 1.5 watt/cm^2^ intensity), and both modalities of US therapy were more effective on pain and functional status than topical application of diclofenac gel [117]. Instead, diclofenac sodium phonophoresis that used US at 1 MHz frequency and 1 watt/cm^2^ intensity was found to be more effective compared to isolated US therapy on pain, stiffness, physical function, and walking time improvement in patients with knee OA [118].

US in continuous mode, 1 W/cm^2^ power, and 1 MHz frequency had comparable efficacy to piroxicam gel phonophoresis (20 mg of piroxicam drug) on pain and total WOMAC score improving in knee OA, without significant differences [119].

The association between US and TENS did not provide additional benefits, this mode of treatment having no significantly different results, compared to piroxicam gel, on pain and total WOMAC score improving in knee OA patients [120].

Compared to US therapy (1 MHz frequency and 1.5 W/cm^2^ intensity), ketoprofen phonophoresis (US at a frequency of 1 MHz, and intensity of 1.5 W/cm^2^ + 100 mg of ketoprofen as 2.5% ketoprofen gel) had no significantly different efficacy on pain relief, WOMAC score, and 15 min walking test improvement [121].

Unfortunately, all studies published to date compared the association of US and topical NSAIDs, namely phonophoresis, with US therapy and none of the studies compared the isolated administration of NSAIDs with US therapy.

## 7. US Therapy Has Favorable Safety Characteristics for Operators, Patients, and Medium

Ionizing radiation, dose accumulation, and risk of cancer are absent for US treatment method. The US waves are poorly transmitted in the air, and protective gear is not needed for US therapy [122]. For US therapy, no cumulative dose has been defined and no adverse event was reported for OA therapeutical protocols. An interesting aspect is that, while NSAIDs can disturb germination, growth and metabolism of plants [55,123,124], exposure to US can be beneficial, increasing germination rate, growth speed, and content of antioxidant compounds [125,126,127].At the same time, potential barriers to completely switching patients with OA from NSAIDs to US therapy have to be taken into account. Completely giving up the administration of NSAIDs in favor of US therapy may be difficult for many patients, due to the large differences between the management of the two forms of treatment. Therefore, NSAIDs are classified as OTC (over-the-counter) drugs, can be administered at home, involve low financial costs, and do not require a significant amount of time for treatment. On the other hand, US therapy requires a medical recommendation, cannot be performed at home, and is financial resources and time consuming. For some patients, US therapy may be considered uncomfortable or even risky in unfavorable epidemiological conditions, as it involves moving away from home and interacting with healthcare professionals. In patients with locomotion difficulties, US therapy may involve additional financial costs that must be analyzed and compared with those of the adverse events of NSAIDs.

## 8. The Limits of This Narrative Review

The most important limitation of this narrative review based on literature data is the lack of comparative studies between the US therapy and NSAIDs in terms of clinical or metabolic effects in OA. To date, there are only a few studies that compare the effectiveness of phonophoresis (US associated with topical NSAIDs) with US therapy and that have proven a similar efficiency or the superiority of phonophoresis.

## 9. Conclusions

Prescription and the consumption of NSAIDs increased enormously in the recent years and will continue to increase, due to the fact that OA, the main disease for which NSAIDs are indicated, has a growing prevalence.

In the absence of alternatives for OA treatment, NSAIDs will continue to pollute the environment and cause genetic, morphological, and functional changes in plants and animals.

US therapy has been shown to have beneficial effects in reducing pain and improving joint function in OA. In addition to NSAIDs, US can reduce cartilage destruction by reducing inflammation and MMP synthesis and by reducing chondrocyte apoptosis, in parallel with increasing collagen and proteoglycan synthesis, without polluting effects on the environment. For these reasons US must be considered as a safe and effective method for OA treatment and a viable alternative to NSAIDs use.

To strengthen this recommendation, new clinical and experimental studies that directly compare the effectiveness of US therapy versus NSAIDs, as well as studies analyzing barriers that may stand in the way of this treatment, are required.

## Data Availability

No new data were created or analyzed in this study. Data sharing is not applicable.
